# Selective lesions of the dorsomedial striatum impair serial spatial reversal learning in rats

**DOI:** 10.1016/j.bbr.2010.02.017

**Published:** 2010-06-26

**Authors:** Anna Castañé, David E.H. Theobald, Trevor W. Robbins

**Affiliations:** a Department of Experimental Psychology and Behavioural and Clinical Neuroscience Institute (BCNI), University of Cambridge, CB2 3EB, Cambridge, United Kingdom; b Department of Neurochemistry and Neuropharmacology, Institut d’Investigacions Biomèdiques de Barcelona (IIBB-CSIC), IDIBAPS, Centro de Investigación Biomédica en Red de Salud Mental (CIBERSAM), Barcelona, Spain

**Keywords:** Behavioural flexibility, Discrimination, Excitotoxic lesion, Learning, Nucleus accumbens, Perseveration, Spatial reversal learning, Striatum

## Abstract

Impairments in reversal learning have been attributed to orbitofrontal cortex (OFC) dysfunction in many species. However, the role of subcortical areas interconnected with the OFC such as the striatum remains poorly understood. This study directly evaluated the contribution of core and shell sub-regions of the nucleus accumbens (NAc), dorsomedial (DMS) and dorsolateral (DLS) striatum to reversal learning of an instrumental two-lever spatial discrimination task in rats. Selective NAc core, DMS and DLS lesions were achieved with microinjections of quinolinic acid and NAc shell lesions with ibotenic acid. Damage to NAc core or shell did not affect retention of a previously acquired instrumental spatial discrimination. In contrast, DLS and DMS lesions produced changes in aspects of discrimination performance such as the latency to collect earned food pellets. Neither NAc core or shell lesions nor DLS lesions affected the main indices of reversal performance. Conversely, DMS lesion rats showed a significant impairment in reversal learning. DMS damage increased the number of errors to reach criteria that were perseverative in nature. The deficit in reversal learning in DMS lesion rats was not associated with an impairment to extinguish instrumental responding. There were no effects on spontaneous locomotor activity. Our data are in agreement with recent work showing that lesions of the medial striatum in marmoset monkeys produce perseverative impairments during a serial visual discrimination reversal task and support the hypothesis that dorsomedial striatal dysfunction contributes to pathological perseveration, which is a common feature of many psychiatric disorders.

## 1 Introduction

Behavioural flexibility, the ability to adjust responding according to changes in strategies, rules or stimulus-reward contingencies, is mediated by the prefrontal cortex (PFC). It is well known that distinct regions of the PFC facilitate different forms of behavioural flexibility. Thus, whereas the medial prefrontal cortex (mPFC) in rodents and lateral PFC in primates play a crucial role in shifting between strategies or attentional sets [13,29,5], the orbitofrontal cortex (OFC) has been identified as a key structure for reversal learning in many species, including humans, monkeys and rodents [23,13,26,21,22,7,10].

Converging evidence suggests that flexible behaviour is not uniquely supported by the PFC but by a larger neural network including prefrontal-basal ganglia circuits. One brain structure that may interact with the PFC to mediate cognitive flexibility includes the striatum ([24], for review see [33,18]). Studies investigating the contribution of the ventral part of the striatum, namely the nucleus accumbens (NAc) in reversal learning are controversial. Lesions of the NAc impaired both initial discrimination and reversal, which renders difficult the interpretation of the latter finding [40,2]. Subsequent studies showed that lesions of the NAc did not disrupt spatial, visual or motor reversal tasks in monkeys [39] or reversal performance in a go-no go odour discrimination paradigm [38] and in a delayed matching task [8] in rats. On the other hand, there is evidence for the participation of the NAc, and in particular its core sub-region, in more complex forms of behavioural flexibility involving changes in strategies or rules [35,17,20].

The dorsal striatum has also been identified as part of the neuroanatomical substrate underlying flexible responding; facilitating both shifts in strategies and response patterns. Studies in rats showed that dorsomedial (DMS) striatal inactivation impairs shifting from a response to visual cue discrimination, and *vice versa* as well as place reversal learning [30,32]. Moreover, cholinergic activity in the dorsomedial striatum contributes to place reversal learning [30,31]. Early work by Divac et al. [14] showed that electrolytic ventrolateral striatal lesions impaired reversal learning in rhesus monkeys. A more recent study in marmoset monkeys has demonstrated that fibre-sparing, excitotoxic lesions of the medial striatum impair reversal learning in a visual serial reversal task [10].

As is the case with the PFC [13], different striatal sub-regions may be implicated in different forms of flexible behaviour. Therefore, the present study was designed to determine the comparative contributions of ventral and dorsal striatum sub-regions to reversal of an instrumental spatial discrimination in rats. Specifically, we report the effects of excitotoxic fiber-sparing lesions of the NAc core and shell as well as DMS and DLS striatum in rats performing serial spatial discrimination reversals. Moreover, we investigated the possibility that any deficits seen in reversal learning were attributable to a failure to extinguish responding on the previously rewarded stimulus using a test of instrumental extinction.

## 2 Materials and methods

### 2.1 Subjects

Fifty-two (Batch 1, *n* = 29; Batch 2, *n* = 23) adult male Lister Hooded rats (Charles River, UK) weighting 280–320 g at the start of experiments were pair-housed under a reversed light cycle (lights on from 19:00 to 07:00 h). Prior to the beginning of training, rats were handled for approximately 5 min daily for 3 days and were put on to a food-restriction schedule (18 g of Purina lab chow per day). Water was available *ad libitum* and testing took place between 12:00 and 16:00 h 7 days per week. The work was carried out under UK Home Office Project licenses (PPL 80/1767 and PPL 80/2234) in accordance with the UK Animals (Scientific Procedures) Act 1986.

### 2.2 Apparatus

Behavioural testing took place within eight operant conditioning chambers (30 cm × 24 cm × 30 cm; Med Associates, Georgia, VT), each enclosed within a sound-attenuating wooden box fitted with a fan for ventilation and masking of extraneous noise. Each chamber was fitted with two retractable levers located on either side of a centrally positioned food magazine, into which an external pellet dispenser could deliver 45 mg sucrose pellets (Noyes dustless pellets; Sandown Scientific, Middlesex, UK), a light-emitting diode (LED), which was positioned centrally above each lever, a magazine light, and a house light. Magazine entry was detected by an infrared photocell beam located horizontally across the entrance. The apparatus was controlled by Whisker control software (www.whiskercontrol.com) and the task was programmed in Visual C++ (v.6).

### 2.3 Behavioural training and testing

**Fig. 1:**
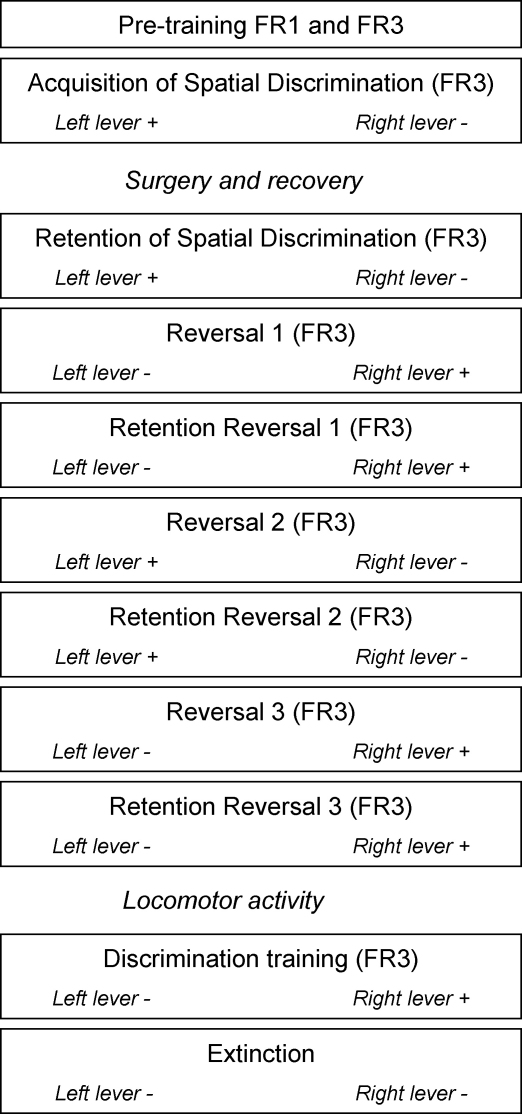
Schematic representation of the behavioural training and testing protocol. The rewarded and unrewarded lever is indicated by the “+” and “−“, respectively. The rewarded lever was counterbalanced across rats.

A schematic representation of the behavioural training and testing protocol is shown in Fig. 1.

*Pre-training*: All rats were initially familiarized with the testing apparatus during 30 min. During this time, the house light was on and the food magazine was loaded with pellets. After the habituation phase, training took place on each lever separately, initially under a fixed ratio 1 (FR1) schedule to a criterion of 50 presses in 15 min, then under a fixed ratio 3 (FR3) schedule to a criterion of 150 presses in 15 min for each lever for two consecutive days. The FR-3 schedule was used to preclude the possibility of reinforcing single, accidental presses on the correct lever. The order of left and right lever presentation was counterbalanced across subjects.

*Acquisition of spatial discrimination*: Training continued with the acquisition of a two-lever discrimination task as previously described [6]. Animals were trained to nose-poke in the central magazine in order to trigger presentation of the retractable levers. Then, three lever presses on only one of these levers would result in reward. Each session lasted 30 min and consisted of a maximum of seven 10-trial blocks. Each trial began with the switching on of the house light. There was a limited hold period of 20 s within which the rat had to nose-poke in the magazine in order to provide the presentation of the two-levers. Lever presentation initiated a 10-s response interval. Failure to respond in either the 20-s limited hold period, or the 10-s response interval resulted in the return to the inter-trial interval (ITI) state until the next trial was scheduled to begin, while the trial was recorded as an omission. Once the rat had responded three times on one of the levers, both levers were retracted and the house light was turned off. Each rat had one training session per day and was trained to a criterion of nine correct trials in one block of 10 trials (binomial distribution *p* < 0.01, likelihood of attaining criterion in a 10-trial block). The completion of nine correct choices out of 10 also implicated the end of the session. Once the criterion was reached, the initial discrimination phase was considered complete, and the animal was returned to the home cage. If the criterion was not achieved, this phase was repeated the next day till criterion achievement.

Then, animals were separated into groups and underwent bilateral NAc core, NAc shell, DMS, DLS lesions or sham surgery. After 10 days of recovery rats were re-tested on discrimination (post-surgical discrimination) and once the criterion was achieved the reversal phase started.

*Serial reversal learning task*: During this phase, rats were trained to serially reverse an instrumental spatial discrimination in a between-session design. A series of three reversals was given, whereby in each reversal the previously correct lever became incorrect and the previously incorrect lever became correct (reversals 1–3). Each reversal session lasted 30 min and consisted of a maximum of seven 10-trial blocks. The learning criterion was the same as in the discrimination phase (nine correct trials in a 10-trial block). Animals usually required more than one session to reach criterion on the reversals. Thus, they received multiple, separate training sessions, the data of which were summed together. Between successive reversals, animals were given a session of up to seven 10-trial blocks where they were required to show retention of the previous reversal phase by reaching the nine of 10 correct criterion in one 10-trial block.

*Locomotor activity*: After completion of the reversal phase, locomotor activity was measured in all rats using 12 computerized photocell beam activity cages. The cages measured 25 cm × 40 cm × 18 cm with two photocell beams dividing the length of the cage into three equal parts. Each photocell beam was positioned 1 cm above the floor of the cage. An Acorn computer (Acorn Computers Ltd., Cambridge, UK) recorded activity during test sessions. The number of beam breaks was recorded over a 60-min period, separated into 5-min time bins. The position of the subjects within the array of the cages was randomized across experimental groups.

*Extinction*: Rats with DMS and DLS lesions and their controls progressed onto an extinction paradigm. Rats were trained on instrumental spatial discrimination (FR3, 70 trials, 30 min session) using the same stimulus contingencies of the last reversal (reversal 3). Once rats had shown accurate performance on two consecutive days (>90% correct trials) extinction commenced the next day. From then on, responses on both levers were not rewarded. Extinction of responding was considered to have occurred after rats performed ≤15 trials on the previously reinforced lever.

*Food pellets intake*: One day after extinction was completed, rats with DMS and DLS lesions and their controls were tested for food consumption in a 30 min test. Briefly, rats were individually housed in standard rat cages where food pellets were freely available in a food cup. The amount of pellets eaten (in grams) was calculated subtracting the weight of the cup containing pellets after the 30 min test from its initial weight.

### 2.4 Surgery

Subjects were divided into groups, matched for their preoperative performance. Animals were anaesthetized and secured in a stereotaxic frame (David Kopf Instruments, Tujunga, CA, USA), fitted with atraumatic ear bars, with the incisor bar set at −3.3 mm relative to the interaural line for a flat skull position. Animals were anaesthetized with inhaled isofluorane, carried in medical oxygen, induced at 5% and maintained at 1–2% concentrations at a flow rate of 2 L/min. Anaesthetic gases were delivered though a nosecone fitted on the incisor bar of the stereotaxic frame (David Koft Instruments). The skill was exposed and a dental drill used to make small holes in the skull above the sites of microinjection. Batch 1 rats received bilateral lesions of the NAc core (*n* = 10), NAc shell (*n* = 11) or sham surgery (NAc core site, *n* = 4; NAc shell site, *n* = 4) as previously described Murphy et al. [27]. Batch 2 rats received bilateral lesions of DMS (*n* = 8) and DLS (*n* = 8) or sham surgery (DMS site, *n* = 4; DLS site, *n* = 3) as previously described Eagle et al. [16]. Different excitotoxins were used to selectively lesion the NAc core, shell and dorsal striatum because of previous experience regarding the relative sensitivity of these regions to excitotoxins (e.g. [27]). For NAc core and dorsal striatum lesions, 0.09 M quinolinic acid (Sigma Aldrich) buffered to pH 7.3–7.4 in 0.1 M phosphate-buffered saline (PBS) was injected according to parameters in Table 1. For shell lesions, 0.06 M ibotenic acid (Sigma–Aldrich), buffered to 7.3 pH in 0.1 M PBS was injected according to parameters in Table 1. Infusions (0.1 μL/min) were made 1 min after lowering the injector into the target region. Coordinates for all surgery were derived using a stereotaxic atlas by Paxinos and Watson [28] using bregma as the origin. Sham operated animals received the same surgical procedure as the lesion groups, except that they were infused with phosphate buffer 0.01 M.

### 2.5 Post-mortem lesion assessment

**Fig. 2:**
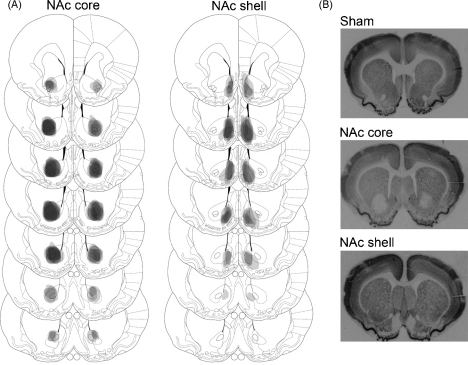
(A) Schematic diagram of a series of coronal sections of the rat brain illustrating the extent of bilateral NAc core and shell lesions (darkness represents coincidence of lesions from different animals). From the top, sections are +2.2, +1.7, +1.6, +1.2, +1.0, +0.7 and +0.48 mm forward of bregma [28]. (B) Photomicrographs of NeuN-stained coronal sections from sham-lesioned, core-lesioned and shell-lesioned animals.

**Fig. 3:**
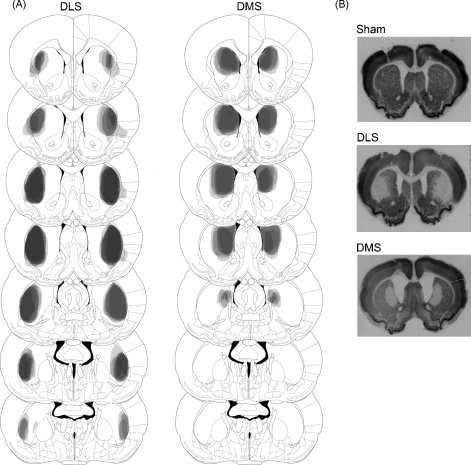
(A) Schematic diagram of a series of coronal sections of the rat brain illustrating the extent of bilateral dorsolateral (DLS) and dorsomedial (DMS) striatum lesions (darkness represents coincidence of lesions from different animals). From the top, sections are +1.7, +1.2, +0.7, +0.2, −0.3, −0.80 and −0.92 mm from bregma [28]. (B) Photomicrographs of NeuN-stained coronal sections from sham-lesioned, dorsolateral-lesioned and dorsomedial-lesioned animals.

After behavioural testing was complete, subjects were anaesthetized with a lethal dose of sodium pentobarbital (1.5 mL/rat, Euthanal, 200 mg/mL; Genus Express, UK) and perfused transcardiacally with 0.01 M PBS followed by 4% paraformaldehyde. The brains were removed and postfixed in 4% paraformaldehyde for 24 h. Prior to being sectioned, brains were transferred to 20% sucrose in 0.01 M PBS as a cryoprotectant and left overnight. Coronal sections of 60 μm were cut on a freezing microtome and processed for immunohistochemistry for the neuron-specific nuclear protein NeuN (Chemicon, Temecula, CA, USA). Specifically, after rinsing in 0.01 M PBS, free-floating sections were incubated overnight at room temperature with a primary mouse anti-NeuN antibody (1:10,000) in a solution containing 0.4% Triton X-100 in 0.01 M PBS. After rinsing, they were incubated for 2 h at room temperature with a secondary biotinylated horse anti-mouse antibody (1:200; Dakopatts, Copenhagen, Denmark) followed by another rinse. The bound antibodies were then visualized by an avidin–biotin–peroxidase complex system (Vectastain ABC Elite Kit, Vector Labs, Burlingame, CA, USA) using 3,3-diaminobenzidine as the chromogen. All sections were mounted onto double-subbed glass slides and coverslipped with DePeX mounting medium (BDH). Lesions were verified by light microscope examination of areas and cell damage was noted by lack of neuronal staining. The extent of lesions was mapped onto standardized sections of the rat brain [28] and is shown in Figs. 2 and 3.

### 2.6 Statistics

Data were analyzed using the STATISTICA package. The main measures of the animals’ ability to learn the discriminations/reversals were: (i) the number of trials to criterion and (ii) the number of errors to criterion (i.e. incorrect trials). Additional measures recorded for each trial were (iii) the type of error, (iv) the latency to collect the reward and (v) the number of omissions. The type of errors was further analyzed as described previously [23,13,9]. Briefly, sessions were classified as perseverative (where responding to the previously correct stimulus was significantly above chance), or learning (where responding to the newly correct stimulus was at, or above chance) by using the two-tailed binomial test. The test was calculated by each rat's day session considering the number of errors and the number of trials data. As an example, ≥44 errors in a 70 trials session indicated perseveration, between 43 and 27 errors was chance performance, and ≤26 errors showed accurate responding to the newly correct lever. Errors during perseverative sessions were considered perseverative errors and so on.

Pre-surgical and post-surgical discrimination data were subjected to one-way ANOVA with lesion group (three levels: sham, core lesion, shell lesion or sham, DLS lesion, DMS lesion) as between-subjects factor of variation. Reversal data were subjected to a repeated measures ANOVA where the between-subjects factor was lesion group and the within-subjects factor the phase (serial reversals: three levels, reversals 1–3; retention of reversals: three levels, retention reversals 1–3) or the type of error (two levels: perseverative and learning). Locomotor activity data were subjected to one-way ANOVA with lesion group as between-subjects factor of variation. Extinction data were analyzed using a repeated measures ANOVA with lesion group as between-subjects factor (three levels: sham, DLS lesion, DMS lesion) and session as within-subjects factor of variation (six levels). Food pellets intake data were subjected to one-way ANOVA with lesion group as between-subjects factor of variation. Where significant interactions were found, they were further explored through separate ANOVAs or *post hoc* comparisons (Newman–Keuls) to establish simple effects. Comparisons were considered statistically significant when *p* < 0.05.

## 3 Results

### 3.1 Histology

Eight of 10 NAc core lesion rats were determined to have appropriate lesions, restricted to the core area of the NAc and not overlapping with the shell region (Fig. 2). Eight of 11 NAc shell rats were determined to have appropriate lesions, restricted to the shell subregion and not overlapping with any other area, and with the lesion focused on the non-lateral shell (Fig. 2). Six of eight DMS lesion rats and all eight DLS lesion rats were determined to have appropriate lesions (Fig. 3). In all cases, there was considerable cell death in the lesioned regions, accompanied by striatal shrinkage. None of the sham lesion rats showed signs of cell damage. One of the NAc shell lesion rats died following surgery. The final numbers (*n*) in each group were: NAc core (*n* = 8), NAc shell (*n* = 8), NAc sham (*n* = 8), DMS (*n* = 6), DLS (*n* = 8), dorsal striatum sham (*n* = 7).

### 3.2 Effects of discrete lesions of the NAc sub-regions (core and shell) on serial spatial reversal learning

*Pre- and post-surgery discrimination*: Before surgery, the three groups of rats did not differ in the number of trials [*F*_(2,20)_ = 0.28; n.s.] or errors [*F*_(2,20)_ = 0.11; n.s.] to reach the performance criterion on the acquisition of instrumemtal spatial discrimination. After surgery, there was no significant difference in the ability of all three groups to remember the previously learned discrimination, and the number of trials [*F*_(2,20)_ = 1.33; n.s.] or errors [*F*_(2,20)_ = 0.28; n.s.] to reach criterion on the retention of spatial discrimination were not significantly different (Table 2).

**Fig. 4:**
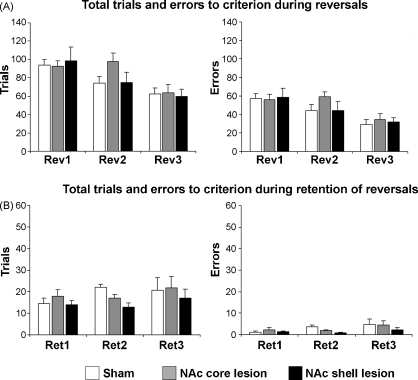
Mean ± SEM of total trials and errors to criterion during (A) reversals and (B) retention of reversals. A series of three reversals (reversal 1–3: Rev1–3) were performed by rats with bilateral lesions of the nucleus accumbens core (NAc core lesion), shell (NAc shell lesion) or control rats (sham). Between successive reversals, animals were given a session to test retention of the previous reversal phase (Ret1–3).

*Serial reversals*: A repeated measures ANOVA (lesion group × reversal phase) of the number of trials revealed no significant main effects of lesion [*F*_(2,20)_ = 0.54; n.s.] or interaction between lesion and reversal [*F*_(4,40)_ = 1.02; n.s.], but a significant main effect of reversal [*F*_(2,40)_ = 12.35; *p* < 0.0001]. Similarly, a repeated measures ANOVA (lesion group × reversal) of the number of errors showed no significant effects of lesion [*F*_(2,20)_ = 0.67; n.s.]. Nor was there an interaction between lesion and reversal [*F*_(4,40)_ = 0.62; n.s.], but there was a significant main effect of reversal [*F*_(2,40)_ = 12.85; *p* < 0.0001], showing that the three groups of rats improved performance during serial reversals equivalently (Fig. 4A).

*Retention of reversal learning*: Rats with bilateral lesions of the NAc core and shell did not show any difference to retain the reversals compared with sham controls. A repeated measures ANOVA (lesion group × retention phase) of the number of trials revealed no significant main effects of lesion [*F*_(2,20)_ = 1.49; n.s.], retention phase [*F*_(2,40)_ = 1.21; n.s.] or interaction [*F*_(4,40)_ = 0.55; n.s.]. A repeated measures ANOVA (lesion group × retention phase) of the number of errors revealed no significant main effects of lesion [*F*_(2,20)_ = 1.29; n.s.], retention phase [*F*_(2,40)_ = 2.44; n.s.] or interaction [*F*_(4,40)_ = 0.61; n.s.] (Fig. 4B).

*Latencies and omissions*: At no point during the discrimination or reversal phases were there any differences between groups in the latency to collect the reward (Table 3) or in the number of omissions (data not shown).

### 3.3 Effects of regional lesions of dorsal striatum (lateral or medial) on serial spatial reversal learning

*Pre- and post-surgery discrimination*: Before surgery, the three groups of rats did not differ in the number of trials [*F*_(2,18)_ = 0.026; n.s.] and errors [*F*_(2,18)_ = 0.14; n.s.] to reach the performance criterion on the acquisition of instrumental spatial discrimination. However, after surgery, the number of trials [*F*_(2,18)_ = 6.84; *p* < 0.01] but not errors [*F*_(2,18)_ = 1.95; n.s.] to reach criteria on retention of discrimination (retention 1) was significantly higher in DLS lesioned rats (*p* < 0.01) and DMS lesioned rats (*p* < 0.05) than in sham animals. After a second discrimination session (retention 2), the groups did not differ in the number of trials [*F*_(2,18)_ = 0.003; n.s.] or errors [*F*_(2,18)_ = 1.17; n.s.] to criterion (Table 2).

**Fig. 5:**
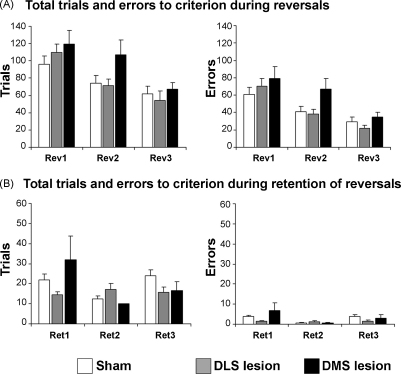
Mean ± SEM of total trials and errors to criterion during (A) reversals and (B) retention of reversals. A series of three reversals (reversal 1–3: Rev1–3) were performed by rats with bilateral lesions of the dorsolateral striatum (DLS lesion), dorsomedial striatum (DMS lesion) or control rats (sham). Between successive reversals, animals were given a session to test retention of the previous reversal phase (Ret1–3).

**Fig. 6:**
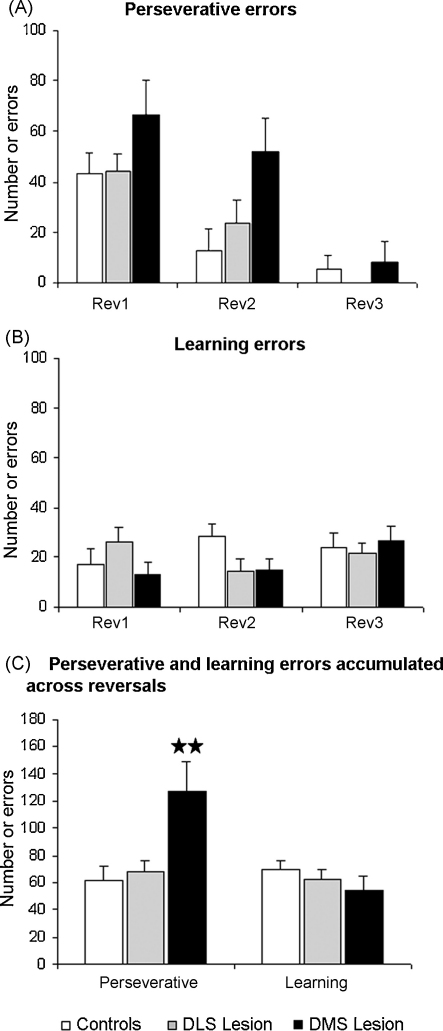
Mean ± SEM of (A) perseverative and (B) learning errors to criterion during serial reversals (Rev1–3) in rats with bilateral lesions of the dorsolateral striatum (DLS lesion), dorsomedial striatum (DMS lesion) or control rats (sham). (C) Error type data collapsed across reversals. Newman–Keuls *post hoc*, ^★^*p* < 0.01 versus sham.

*Serial reversals*: A repeated measures ANOVA (lesion group × reversal phase) of the number of trials to criterion revealed no significant main effects of lesion [*F*_(2,18)_ = 2.45; n.s.] or interaction between lesion and reversal [*F*_(4,36)_ = 0.81; n.s.], but a significant main effect of reversal [*F*_(2,36)_ = 16.08; *p* < 0.0001]. Concerning the number of errors, which is one of the most sensitive measures of animals’ performance on the spatial discrimination and reversal task, a repeated measures ANOVA (lesion group × reversal) showed a significant main effect of lesion [*F*_(2,18)_ = 3.90; *p* < 0.05] and reversal [*F*_(2,36)_ = 21.22; *p* < 0.0001], but there was not a significant interaction between these factors [*F*_(4,36)_ = 0.77; n.s.] (Fig. 5A). When we further explored how the different type of errors (classified according to [23,13,9]) were affected by the lesions, we observed a significant main effect of lesion [*F*_(2,18)_ = 6.36; *p* < 0.01] and reversal [*F*_(2,36)_ = 21.82; *p* < 0.0001], but no interaction between these [*F*_(4,36)_ = 1.04; n.s.] for the number of perseverative errors. However, there were no significant effects of lesion [*F*_(2,18)_ = 0.88; n.s.], reversal [*F*_(2,36)_ = 0.87; n.s.] or interaction [*F*_(4,36)_ = 1.57; n.s.] for the number of learning errors (Fig. 6A and B). Across reversals, DMS lesion rats made more perseverative errors than controls, an effect that was clearly observed when expressing the two type of errors (perseverative and learning) collapsed across all three reversals (Fig. 6C). A repeated measures ANOVA (lesion group × error type) showed a significant main effect of lesion [*F*_(2,18)_ = 3.92; *p* < 0.05], error type [*F*_(1,18)_ = 5.32; *p* < 0.05] and interaction between lesion and error type [*F*_(2,18)_ = 5.68; *p* < 0.05]. *Post hoc* analysis revealed that DMS lesioned rats made significantly more perseverative errors than controls (*p* < 0.01) (Fig. 6C). No differences were noted in learning errors.

*Retention of reversals*: Rats with bilateral lesions of the DMS and DLS exhibited similar retention of the reversal learning discrimination to that of sham-operated controls. A repeated measures ANOVA (lesion group × retention phase) of the number of trials revealed no significant main effects of lesion [*F*_(2,18)_ = 1.07; n.s.] and interaction [*F*_(4,36)_ = 2.40; n.s.] but a significant main effect of retention phase [*F*_(2,36)_ = 3.45; *p* < 0.05]. Similarly, A repeated measures ANOVA (lesion group × retention phase) of the number of errors revealed no significant main effects of lesion [*F*_(2,18)_ = 2.63; n.s.], and interaction [*F*_(4,36)_ = 1.55; n.s.], but a significant main effect of retention phase [*F*_(2,36)_ = 4,27; *p* < 0.05] (Fig. 5B).

*Latencies and omissions*: DLS lesion rats took significantly longer to collect earned food pellets than sham-operated control rats in all post-surgical behavioural tests (Table 3). The latency to collect the reward was also prolonged in rats with DMS lesions. However, this last group of rats recovered and the latency reached a level similar to that of controls by the end of behavioural testing (from the retention of reversal 2 to retention of reversal 3) (Table 3).

A significant lesion effect was also observed in the number of omissions during the first post-surgical discrimination (mean ± SEM in sham, 0.3 ± 0.2; DLS, 26.1 ± 4.4; DMS, 5.7 ± 3.1) [*F*_(2,17)_ = 20.05; *p* < 0.001]. DLS lesion rats made more omissions than controls (*p* < 0.001) but no other significant differences between groups were observed in the number of omissions during the reversals and their retention (data not shown).

*Body weight*: The mean weight (in g) of the DLS lesion group was significantly lower than that of the control group (*p* < 0.05) (mean weight at the end of the experiment period ± SEM; sham, 398.6 ± 11.6; DMS, 376.2 ± 7.9; DLS, 358.1 ± 8.3).

### 3.4 Effects of regional lesions of NAc and dorsal striatum on spontaneous locomotor activity

**Fig. 7:**
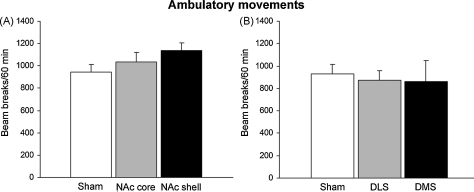
Mean ± SEM of number of beam breaks during a 60-min test session as a measure of ambulatory activity in rats with bilateral lesions of (A) the nucleus accumbens core (NAc core), nucleus accumbens shell (NAc shell) or controls (sham), and (B) dorsolateral striatum (DLS), dorsomedial striatum (DMS lesion) or controls (sham).

Rats with regional NAc (core and shell) and dorsal striatum (DLS and DMS) lesions showed similar levels of spontaneous locomotor activity (ambulatory movements) as control subjects, as measured by the number of beam breaks during the 60-min test session (NAc: *F*_(2,20)_ = 1.50, n.s.; dorsal striatum: *F*_(2,18)_ = 0.08, n.s.; Fig. 7).

### 3.5 Effect of regional lesions of the dorsal striatum on responding in extinction

**Fig. 8:**
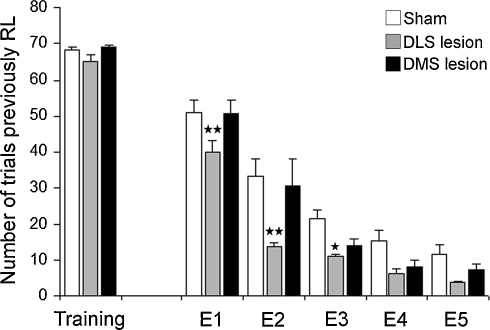
Mean ± SEM of number of trials in the previously reinforced lever (RL) during the last day of training (T2) and extinction sessions 1–5 (E1–E5) in rats with bilateral lesions of dorsolateral striatum (DLS), dorsomedial striatum (DMS lesion) or controls (sham). Newman–Keuls *post hoc*, ^★^*p* < 0.05; ^★★^*p* < 0.01 versus sham.

DLS lesion rats extinguished instrumental responding significantly faster than controls. A repeated measures ANOVA (lesion group × session) of the number of responses on the previously reinforced lever showed a significant main effect of lesion [*F*_(2,17)_ = 8.24; *p* < 0.01], session [*F*_(5,85)_ = 307.40; *p* < 0.0001] and interaction [*F*_(10,85)_ = 2.21; *p* < 0.05]. DLS lesion rats made fewer responses than controls in the previously reinforced lever during extinction sessions 1 (*p* < 0.01), 2 (*p* < 0.01) and 3 (*p* < 0.05) (Fig. 8).

### 3.6 Food pellets intake in dorsal striatum lesion rats and controls during a 30 min test

One-way ANOVA showed no differences on the amount of food (in g) that DMS, DLS lesioned rats and controls ate in a 30 min test where pellets were freely available (mean grams of pellets eaten ± SEM; sham, 15.6 ± 1.1; DMS, 12.9 ± 2.1 and DLS, 13.5 ± 1.4).

## 4 Discussion

This study has demonstrated differential effects of selective lesions of ventral and dorsal striatum sub-regions on serial reversal learning of an instrumental spatial discrimination task in rats. Damage to DMS induced perseverative responding in reversal learning whereas NAc core, shell or DLS lesions did not. Despite the impairment in reversal learning, DMS lesion rats extinguished instrumental responding at the same rate as sham-operated controls. Conversely, DLS lesions facilitated extinction, but did not impair reversal learning.

### 4.1 Probable lack of role of NAc core and shell in spatial reversal learning

Previous studies demonstrated that the NAc plays an important role on the acquisition of instrumental contingencies [3]. Here, we observed that NAc core and shell lesions affected neither the retention of a previously learned instrumental spatial discrimination nor its reversal. Moreover, the latency to collect the reward, the number of omissions and locomotor activity were also unaffected by NAc lesions.

Previously published data in rhesus monkeys using a spatial, visual or motor reversal task [39], or in rats, the reversal of a go-no go odor discrimination [38] or a delayed matching task [8] all show that ventral striatum lesions did not reliably impair reversal learning performance. However, the possible role of NAc sub-regions in reversal learning has not previously been explored. Here, we report that selective lesions of the NAc core and shell did not affect the reversal of a spatial discrimination in rats, suggesting that neither sub-region is critically involved in this form of behavioural flexibility.

In contrast, recent studies have shown that NAc mediates behavioural flexibility during switching of general rules, strategies or attentional sets [17,20]. Floresco et al. [17] reported a dissociable contribution of NAc core and shell to behavioural flexibility during set-shifting. Inactivation of the NAc core by infusion of the GABA agonists baclofen and muscimol impaired changing from response to visual cue discrimination and vice versa in a maze-based strategy set-shifting task. This effect was attributable to an inability of rats to acquire or maintain the new strategy. Conversely, similar manipulations of the NAc shell either did not impair or even improved shift performance [17]. Moreover, in a recent study, Haluk and Floresco [20] explored the possible contribution of ventral striatal dopamine in behavioural flexibility using dopamine receptor agonists and antagonists. D1, but not D2, receptor blockade in the NAc core retarded the ability to shift between discrimination strategies, suggesting that D1 receptor tone in the NAc facilitates this process. On the contrary, neither D1 nor D2 receptor antagonism affected reversal learning. In the same study, stimulation of D2 but not D1 receptors in the NAc core produced a generalized deficit in behavioural flexibility impairing both strategy set-shifting and reversal learning processes. It is notable that damage to the NAc (present study, [39,8,38]) does not disrupt reversal learning performance but D2 stimulation in the NAc core does [20]. The latter authors argued that D2 receptor stimulation in the NAc may produce an abnormal stimulation of these receptors which impairs reversal learning performance. However, the absence of an effect of the D2 receptor antagonist led these authors to conjecture that other striatal sub-regions may be implicated in reversal performance. Thus our own recent data showing that systemic administration of quinpirole impaired reversal learning of a spatial discrimination in rats through a D2-like receptor mechanism [6] may possibly be explained by effects of quinpirole at other striatal sites as well as in the NAc core sub-region. These observations may be of interest in the context of neuropsychiatric disorders such as schizophrenia, where dopamine receptor over stimulation is likely to be present [19,1].

### 4.2 Possible role of dorsomedial and dorsolateral striatum lesions in reversal learning

Selective excitotoxic lesions of the DMS induced inflexible behaviour in rats during serial reversals of an instrumental spatial discrimination. This effect was exclusively dependent on the medial sector of the dorsal striatum since DLS damage did not impair reversal performance. In contrast, there were no deficits in the retention of reversals in any group of rats. Several aspects of behavioural performance, though not spontaneous locomotor activity, were also modified after both medial and lateral dorsal striatum damage.

Lesions to the medial and lateral sectors of the dorsal striatum affected the ability to retain a previously learned spatial discrimination, increasing the number of successful trials but not errors to achieve criteria during the first post-surgical discrimination. DLS lesion rats also showed an increased number of omissions during the retention of discrimination, which may account for the increased number of trials. Therefore, DLS, DMS lesion rats and controls were given a second post-surgical discrimination to ensure that performance was similar in all groups before the initiation of the reversal phase.

DMS but not DLS lesions impaired serial reversal learning of an instrumental spatial discrimination. This deficit was apparent in early stages of the reversal phase but not in the last reversal, suggesting that the contribution of DMS may decrease with task experience. A similar region of the DMS mediates action–outcome contingency learning [4]. However, a major part of DMS lesioned rats reversal deficits appeared to be a failure to suppress perseverative responding for the original contingency.

Specific reversal learning deficits have been previously observed in relation to dorsal striatum dysfunction (see [33] for review). Electrolytic lesions of the caudate–putamen induced perseverative tendencies in rats during spatial reversals [24]. Recently, marmoset striatal lesions, including both the medial caudate nucleus and the NAc, increased perseveration during the serial reversal of a visual discrimination [10]. DMS lesions in rats (present study) and medial striatum lesions in monkeys [10] produced perseverative impairments similar to those observed after lesions of the OFC [7,10]. Taking into account that one major recipient of OFC projections is the striatum [37,36], the results exposed here raise the possibility that OFC and DMS form a functional fronto-striatal circuit critical for the mediation of behavioural flexibility during reversal learning.

Despite the perseverative impairments observed after medial striatum lesions, temporary inactivation of the DMS with bupivacaine infusions impaired place reversal, although the deficits were attributable to a failure to engage or maintain the new response [30,33]. Experimental differences such as the training protocol for discrimination learning, the timing of dorsomedial striatum inactivation/lesion, as well as the size of the lesion or inactivated zone may account for differences in findings. Previous authors have also identified DMS acetylcholine as being contributory to place reversal learning [30,41,31]. It appears likely that different neurotransmitters in the DMS may influence distinct processes that enable reversal learning, such as the inhibitory control of previously reinforced responses and the engagement of new stimulus-reward associations. Pharmacological studies have clearly implicated dopaminergic mechanisms in reversal learning [11,25,6,20]. Therefore, more direct investigations of the contribution of local dopaminergic activity within the DMS to reversal learning would be of interest.

Dorsal striatum lesions impaired other aspects of performance, namely the latency to collect the food pellets. Although present in both lesion groups, DLS-lesioned rats took significantly longer to collect reward food pellets and were consistently lower in weight throughout the experiment. However, decreased motivation for food in striatal lesioned rats is unlikely to explain the present findings since neither medial nor lateral lesions of the dorsal striatum had any effect on the final breaking point of a progressive ratio schedule for food reinforcement [15]. Moreover, the amount of food (in g) that rats consumed during a 30 min test where pellets were freely available was not significantly different across the groups. Similar increases in latencies and weight changes were previously described after medial and lateral dorsal striatum lesions [15] and may reflect the involvement of dorsal striatum in particular aspects of behaviour.

### 4.3 Effects of dorsomedial and dorsolateral striatum lesions on responding in extinction

Because reversal learning impairments in DMS lesion rats may have been due to a failure to inhibit a prepotent instrumental response, we sought to test whether the same lesions affected the extinction of instrumental responding following reinforcement omission. Extinction performance was comparable between DMS lesion rats and controls. Other studies have also observed that increased perseveration in reversal learning is not necessarily associated with resistance to extinction [10,42], suggesting that distinct neuroanatomical substrates may govern behavioural extinction and reversal learning. In this regard, we observed that despite the unaltered performance in reversal learning, DLS lesioned rats showed a marked facilitation of extinction of responding to the previously reinforced lever. Although DLS inactivation appeared to enhance the rats’ sensitivity to changes in action–outcome contingency during instrumental conditioning [43], further studies are needed to clarify whether facilitation of extinction in these rats is due to increased sensitivity to non-reward or to reduced control by conditioned reinforcement (e.g. see [12]). We did not investigate effects of NAc lesions on extinction in this study, but it is of interest that selective DA depletion from the NAc but not the dorsal striatum retarded resistance to extinction in a conditional visual discrimination task [34].

## 5 Conclusion

In summary, the present work has demonstrated that neither the NAc (core or shell sub-regions) nor the dorsolateral striatum has a critical role in mediating reversal learning of a spatial discrimination, whereas dorsomedial striatal lesions do selectively impair this ability. Moreover, dorsolateral, but not dorsomedial striatal lesions reduce resistance to extinction thus dissociating reversal learning from extinction. These results enhance our understanding of how distinct striatal sub-regions mediate different forms of behavioural flexibility.

## Figures and Tables

**Table 1:** Injection parameters for selective lesions of nucleus accumbens and dorsal striatum sub-regions.

Lesion area	Injections per site	Excitotoxin	Coordinates			Injection		Diffusion
			AP	L	DV	Vol (μl)	Time (min:s)	Time (min:s)
NAc core	1	0.09 M Quinolinic acid	+1.2	±1.8	−7.1	0.30	3:00	3:00

NAc shell	3	0.06 M Ibotenic acid	+1.6	±1.1	−7.9	0.16	1:30	1:30
					−6.9	0.10	1:00	1:00
					−6.4	0.10	1:00	1:00
								

DLS	4	0.09 M Quinolinic acid	−0.3	±3.6	−6.5	0.175	1:40	1:40
					−6.0	0.175	1:40	1:40
			+0.7	±3.6	−6.0	0.175	1:40	1:40
					−5.5	0.175	1:40	1:40

DMS	4	0.09 M Quinolinic acid	+0.2	±2.0	−5.5	0.175	1:40	1:40
					−4.5	0.175	1:40	1:40
			+1.2	±2.0	−5.5	0.175	1:40	1:40
					−4.5	0.175	1:40	1:40

NAc, nucleus accumbens; DLS, dorsolateral striatum; DMS, dorsomedial striatum. AP, anteroposterior; L, lateral from midline; DV; dorsoventral. DV coordinates were taken from the skull surface.

**Table 2:** Pre- and post-surgery discrimination performance.

Group	Pre-surgery discrimination		Post-surgery discrimination			
	Acquisition		Retention 1		Retention 2	
	Trials	Errors	Trials	Errors	Trials	Errors
NAc sham	44.3 ± 5.9	13.0 ± 2.1	13.5 ± 3.6	2.3 ± 1.8	–	–
NAc core lesion	47.9 ± 5.2	14.0 ± 1.4	20.5 ± 4.7	2.1 ± 0.7	–	–
NAc shell lesion	42.0 ± 5.4	12.4 ± 3.5	12.1 ± 3.0	1.0 ± 0.8	–	–

DS sham	48.3 ± 12.7	13.3 ± 3.1	14.0 ± 3.0	2.9 ± 2.4	13.3 ± 1.8	0.4 ± 0.3
DLS lesion	47.9 ± 7.4	15.5 ± 4.0	45.4 ± 6.5**	7.6 ± 2.3	13.3 ± 3.0	0.1 ± 0.1
DMS lesion	50.8 ± 7.8	15.8 ± 3.7	37.7 ± 8.9*	9.7 ± 2.7	13.5 ± 1.7	0.7 ± 0.3

Number of trials and errors made to reach criterion on the acquisition of spatial discrimination (pre-surgery) and the retention of discrimination (post-surgery) for rats with regional lesions of the nucleus accumbens, NAc (core, shell), dorsal striatum, DS (dorsolateral striatum, DLS; dorsomedial striatum, DMS), and controls (sham lesions). Data are expressed as mean ± SEM.

* *p* < 0.05 (*post hoc* Newman–Keuls) versus sham.

** *p* < 0.01 (*post hoc* Newman–Keuls) versus sham.

**Table 3:** Latency to collect the pellet during discrimination (A) and reversal (B) phases.

Group	Discrimination phase		
	Acquisition	Retention 1	Retention 2
A			
NAc sham	768 ± 45	565 ± 39	–
NAc core	911 ± 88	720 ± 111	–
NAc shell	622 ± 71	481 ± 58	–

DS sham	823 ± 55	562 ± 57	537 ± 56
DLS	903 ± 67	1340 ± 162**	1051 ± 173*
DMS	876 ± 78	996 ± 125*	635 ± 76

Group	Reversal phase					
	Rev1	Ret1	Rev2	Ret2	Rev3	Ret3
B						
NAc sham	781 ± 51	560 ± 39	638 ± 62	538 ± 41	553 ± 24	480 ± 38
NAc core	825 ± 80	605 ± 57	689 ± 73	606 ± 78	564 ± 61	485 ± 32
NAc shell	660 ± 67	533 ± 94	618 ± 50	464 ± 36	477 ± 35	408 ± 20

DS sham	768 ± 63	531 ± 45	571 ± 67	436 ± 26	475 ± 37	434 ± 35
DLS	1437 ± 171*	1102 ± 123*	1086 ± 117**	1059 ± 170**	1042 ± 167*	892 ± 123*
DMS	1355 ± 204*	957 ± 180*	865 ± 91*	586 ± 60	770 ± 121	615 ± 89

Mean ± SEM of latencies (milliseconds) to collect the reward during (A) the acquisition of spatial discrimination (pre-surgery) and the retention of discrimination (post-surgery; retention 1, retention 2), as well as (B) during the reversal phase (reversal 1–3, Rev1–3; retention of reversal 1–3; Ret1–3) in rats with regional lesions of the nucleus accumbens, NAc (core, shell), dorsal striatum, DS (dorsolateral striatum, DLS; dorsomedial striatum, DMS), and controls (sham lesions).

* *p* < 0.05 (*post hoc* Newman–Keuls) versus sham.

** *p* < 0.01 (*post hoc* Newman–Keuls) versus sham.
